# Predictors of late presentation and advanced HIV disease among people living with HIV in Oman (2000–2019)

**DOI:** 10.1186/s12889-021-12048-1

**Published:** 2021-11-06

**Authors:** Ali Elgalib, Samir Shah, Adil Al-Wahaibi, Zeyana Al-Habsi, Maha Al-Fouri, Richard Lau, Hanan Al-Kindi, Bader Al-Rawahi, Seif Al-Abri

**Affiliations:** Directorate General for Disease Surveillance and Control, Ministry of Health, Muscat, Oman

**Keywords:** HIV/AIDS, Late presentation, Oman, Advanced HIV, Predictors

## Abstract

**Background:**

The aim of this study was to determine the proportions and predictors of late presentation (LP) and advanced HIV disease (AD) in Oman. LP and AD were defined as presenting with a baseline CD4 count of < 350 and < 200 cells/mm3, respectively.

**Methods:**

We conducted a retrospective database analysis of the National HIV Surveillance System to identify Omani people (≥ 13 years old) who were diagnosed with HIV in the period between January 2000 and December 2019 and had a documented baseline CD4 cell count. We calculated the rates and trend over time of LP and AD. A logistic regression was carried out to determine the predictors of LP and AD.

**Results:**

A total of 1418 patients, who were diagnosed with HIV in the period from January 2000 to December 2019, were included; 71% were male and 66% were heterosexuals. The median (IQR) age at diagnosis was 33 (25–39) years. Overall, 71% (95% CI: 68–73) and 46% (95% CI: 44–49) of patients had LP and AD at presentation, respectively. The LP percentage decreased from 76% in 2000–2004 to 69% in 2015–2019; AD percentage decreased from 57 to 46% over the same period. The proportions of men with LP and AD were higher than women (74% vs. 62 and 50% vs. 36%, respectively). The percentages of persons with LP among people aged 13–24, 25–49, and ≥ 50 years were 65, 71, and 84%, respectively. The proportions of persons with AD among people aged 13–24, 25–49, and ≥ 50 years were 39, 46, and 65%, respectively. Logistic regression showed that male sex, older age, having an “unknown” HIV risk factor, and living outside Muscat were independent predictors of AD. Male sex also independently predicted LP.

**Conclusions:**

This analysis indicates that a significant proportion of new HIV cases in Oman continue to present late. This study identified patient subgroups at greatest risk of late HIV diagnosis such as men and older people. Targeted interventions and greater efforts to scale up HIV testing services in Oman are needed.

**Supplementary Information:**

The online version contains supplementary material available at 10.1186/s12889-021-12048-1.

## Background

Late HIV diagnosis is associated with increased morbidity and mortality [1, 2], greater onward HIV transmission [3, 4], and high cost of treatment and care [5]. For example, two UK-based studies have indicated that people who are diagnosed at a late stage of their HIV infection (CD4 count < 350 cells/mm3) are 3.5 times more likely to die and have a ten-fold risk of death within 12 months of diagnosis compared to patients who are diagnosed early [1, 2]. Moreover, Marks G et al. [3] reported that people who are unaware of their HIV infection are more likely to engage in high risk-taking behaviour compared to those who are aware of their HIV diagnosis. Conversely, individuals who know their HIV serostatus can modify their risk behaviours and access treatment, both of which can help reduce onward HIV transmission [3, 4].

In 2011, the European Late Presenter Consensus Working Group published their definitions for late HIV diagnosis. Late presentation (LP) was defined as the presence of either an AIDS-related condition regardless of CD4 cell count or a CD4 count of < 350 cells/mm3 at presentation for care. Advanced HIV disease (AD) was defined as the presence of either an AIDS-related illness regardless of CD4 cell count or a CD4 count of < 200 cells/mm3 at presentation [6]. Late HIV diagnosis could be attributed to barriers to HIV testing related to policy, provider, and patient [7–9]. For instance, low-risk perception and fears around stigma, discrimination, confidentiality breaches, and criminalisation of particular risk behaviours are some of the patient-related barriers to HIV testing [7, 8]. In addition, providers have reported operational barriers such as lack of time, the need for training, and concerns about giving results to and follow-up of HIV positive individuals [8]. Moreover, punitive laws deter those most at risk of HIV, such as sex workers, men who sex with men (MSM) and people who inject drugs (PWID), from accessing HIV testing services [9].

The Sultanate of Oman is located in the Arabian Peninsula, with a total population of 4,601,706 of whom 2,022,470 (44%) are non-Omanis [10]. The HIV incidence and prevalence are low in Oman [11, 12]; the Joint United Nations Programme on HIV and AIDS (UNAIDS) data show that HIV incidence (0.07 per 1000 adults) and prevalence (0.2 per 1000 adults) in Oman were stable in 2010–2019 [12]. The Omani National AIDS Programme (NAP) was formed in 1996. There are currently 14 public treatment centres in the country. All treatment centres have access to CD4 testing, with a national central public health laboratory (CPHL) located in Muscat that is responsible for performing HIV viral load (VL) and genotypic resistance testing.

Recently published data about the 2018 HIV care cascade in Oman revealed that the quality of HIV care has significantly improved over recent years; high rates of retention in care, ART coverage, and viral suppression were demonstrated. However, late HIV diagnosis was highlighted as one of the main challenges for the HIV programme in Oman; the median (interquartile range [IQR]) CD4 cell count at diagnosis, for the 1532 people living with HIV as of December 2018, was 244 cells/mm3 (106–408 cells/mm^3^) [13]. In this study, we used data from the national HIV registry to further characterise the late HIV diagnosis in the country. We sought to determine the proportions, trend over time and predictors of LP and AD among individuals diagnosed with HIV in Oman between 2000 and 2019.

## Methods

### Population

We included Omani people (≥ 13 years old) who were diagnosed with HIV in the period from January 2000 to December 2019 and had a documented baseline CD4 cell count (the first measurement after diagnosis and before ART initiation).

### Outcome measures

LP and AD were defined as presenting with a baseline CD4 cell count of < 350 and < 200 cells/mm^3^, respectively.

### Data collection

Patients were identified from a national HIV surveillance system. Data included sex, age, HIV risk factor (heterosexual, MSM, other and unknown), reason for HIV testing (HIV-related symptoms, HIV contact, antenatal screening, patient’s request and other), baseline CD4 cell count, year of HIV diagnosis, marital status, and residence. HIV risk factor “other” included PWID, vertical transmission, and blood transfusion. HIV mode of acquisition was categorised as “unknown” when the patient declined to disclose their HIV risk behaviour.

### Data analysis

We conducted a retrospective database analysis to calculate the rates of LP and AD. Descriptive univariate analysis for sociodemographic and clinical parameters was performed; categorical variables were reported as frequency and percentage, and continuous variables were reported as medians and interquartile ranges. We carried out univariate logistic regression to assess the unadjusted association (crude odds ratio [cOR]) between the predictor and outcome variables. We also performed multivariate logistic regression to determine the independent predictors of LP and AD; we adjusted models for sex, age at diagnosis (13–24, 25–49 and ≥ 50 years), HIV risk group, the reason for HIV testing, year of HIV diagnosis (2000–2004, 2005–2009, 2010–2014 and 2015–2019), residence (Muscat and outside Muscat) and marital status (single, married and divorced/widowed). The multivariate model was theory-based; therefore, we included all predictor variables in the multivariate model irrespective of their statistical significance in the univariate analysis. Adjusted odds ratio (aOR) and cOR were reported at a 95% confidence interval (CI). We analysed data using Microsoft Excel 2016 and R Software (R Foundation for Statistical Computing, Vienna, Austria) version 3.6.0 (2019).

### Ethical considerations

We used programme data collected for routine patient care that was submitted to the NAP. Permission to use the data was obtained from the Directorate General for Disease Surveillance and Control at the ministry of health who deemed the study as a public health programme evaluation according to national regulations. Therefore, institutional review board approval and informed consent were not sought.

## Results

A total of 2215 individuals (≥ 13 years old) were diagnosed with HIV in 2000–2019. Women were more likely to be tested for CD4 cell count at presentation compared with men (74% vs. 61%) (Supp 1). The proportions of patients who were tested for baseline CD4 cell count among patients aged 13–24, 25–49, and ≥ 50 years were 70, 65 and 46%, respectively (Supp 1). Patients without CD4 count at baseline (*n* = 797) were excluded from further analysis. Of the 1418 individuals with a baseline CD4 cell count test, 71% were male and 66% self-identified as heterosexuals. The median (IQR) age at diagnosis and CD4 cell count at presentation were 33 (25–39) years 222 (79–388) cells/mm^3^, respectively.

**Fig. 1:**
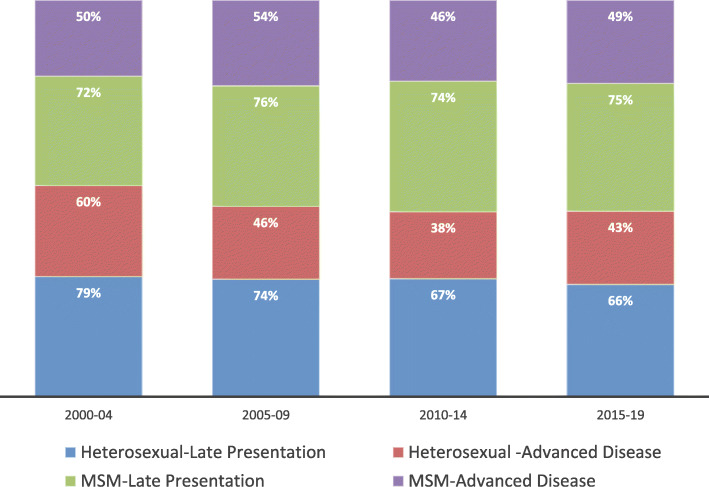
Percentages of advanced disease and late presentation by HIV risk factor, 2000–2019

Overall, 71% (95% CI: 68–73) and 46% (95% CI: 44–49) of patients had LP and AD at presentation, respectively (Table 1). The proportions of men with LP and AD were higher than women (74% vs. 62 and 50% vs. 36%, respectively). The percentages of persons with LP among people aged 13–24, 25–49, and ≥ 50 years were 65, 71, and 84%, respectively. The proportions of persons with AD among people aged 13–24, 25–49, and ≥ 50 years were 39, 46, and 65%, respectively. Compared to heterosexuals, patients in the “unknown” HIV risk category had higher proportions of LP (79% vs. 69%) and AD (64% vs. 43%). Overall, the LP percentage decreased from 76% in 2000–2004 to 69% in 2015–2019; AD percentage decreased from 57 to 46% over the same period. In 2000–2004, higher proportions of heterosexuals presented with LP (79% vs. 72%) and AD (60% vs. 50%) compared to MSM. By contrast, higher proportions of MSM met the definition for LP (75% vs. 66%) and AD (49% vs. 43%) compared to heterosexuals in 2015–2019 (Fig. 1).

**Table 1:** Advanced HIV disease and late presentation in Oman, 2000–2019 (*N* = 1418)

Characteristic (All cases)	Advanced Disease n (%)	Late Presentation n (%)
Total (*N* = 1418)	654 (46)	1001 (71)
Sex		
Male (*N* = 1003)	503 (50)	742 (74)
Female (*N* = 415)	151 (36)	259 (62)
Age at diagnosis category (years)		
13–24 (*N* = 308)	120 (39)	200 (65)
25–49 (*N* = 982)	450 (46)	693 (71)
≥ 50 (*N* = 128)	84 (65)	108 (84)
HIV Risk factor		
Heterosexual (*N* = 934)	402 (43)	642 (69)
Men who have sex with men (*N* = 296)	144 (47)	220 (74)
Other (*N* = 32)	8 (25)	16 (50)
Unknown (*N* = 156)	100 (64)	123 (79)
Reason for HIV testing		
HIV-related symptoms (*N* = 417)	264 (63)	350 (84)
HIV contact (*N* = 143)	49 (34)	93 (65)
Antenatal screening (*N* = 108)	22 (20)	46 (43)
Patient request (*N* = 90)	28 (31)	48 (53)
Other (*N* = 314)	98 (31)	187 (60)
Year of diagnosis		
2000–2004 (*N* = 95)	54 (57)	72 (76)
2005–2009 (*N* = 239)	118 (49)	180 (75)
2010–2014 (*N* = 481)	203 (42)	335 (70)
2015–2019 (*n* = 603)	279 (46)	414 (69)
Region of residence		
Muscat (*N* = 392)	171 (44)	270 (69)
Outside Muscat (*N* = 1026)	483 (47)	731 (71)
Marital status		
Married (*N* = 728)	317 (44)	500 (69)
Single (*N* = 519)	242 (47)	365 (70)
Divorced/widowed (*N* = 124)	69 (56)	97 (78)

### Factors associated with AD

The univariate analysis (Table 2) showed that male sex compared to female sex (OR 1.76 (1.39–2.23), being aged ≥50 years compared to being aged 13–24 years (OR 2.99 (1.94–4.6), having an “unknown” HIV risk factor compared to heterosexuals (OR 2.36 (1.66–3.36), and being divorced or widowed compared to being married (OR 1.63 (1.11–2.39) significantly increased the odds of presenting with AD. Testing for HIV for reasons other than HIV-related symptoms significantly decreased the odds of AD (OR 0.3 (0.2–0.45) for HIV contact; OR 0.15 (0.09–0.25) for antenatal screening; OR 0.26 (0.16–0.43) for patient request). The multivariate analysis (Table 2) revealed that men compared to women (aOR 1.4, 95% CI: 1.02–1.92) and those aged ≥50 years compared to persons aged 13–24 years (aOR 1.89, 95% CI: 1.15–3.11) had significantly higher odds of presenting with AD. Similarly, being in the “unknown” HIV risk category compared to heterosexual (aOR: 1.78, 95% CI: 1.19–2.68) and living outside Muscat compared to residing in Muscat (aOR: 1.31, 95% CI: 1.02–1.7) were independent predictors of AD. Compared to people testing for HIV due to HIV related symptoms, those testing because of being in contact with an HIV positive individual (aOR: 0.38, 95% CI: 0.25–0.59) or antenatal screening (aOR: 0.24, 95% CI: 0.13–0.42) or patient’s request (aOR: 0.28, 95% CI: 0.17–0.46) or “other” reason (aOR: 0.28, 95% CI: 0.2–0.38) were significantly less likely to present with AD.

**Table 2:** Factors associated with advanced HIV disease in Oman, 2000–2019 (*N* = 1418)

Variable	Crude OR (95% CI)	***P*** value	Adjusted OR (95% CI)	***P*** value
Sex				
Female	Reference		–	
Male	**1.76 (1.39–2.23)**	< .001	**1.4 (1.02–1.92)**	0.035
Age at diagnosis category (years)				
13–24	Reference		–	
25–49	**1.33 (1.02–1.72)**	0.035	1.2 (0.89–1.61)	0.236
≥ 50	**2.99 (1.94–4.6)**	< .001	**1.89 (1.15–3.11)**	0.012
HIV Risk factor				
Heterosexual	Reference		–	
MSM	1.25 (0.97–1.63)	0.091	1.01 (0.74–1.37)	0.954
Other	**0.44 (0.2–0.99)**	0.048	0.52 (0.22–1.23)	0.137
Unknown	**2.36 (1.66–3.36)**	< .001	**1.78 (1.19–2.68)**	**0.005**
Reason for HIV testing				
HIV-related symptoms	Reference		–	
HIV contact	**0.3 (0.2–0.45)**	< .001	**0.38 (0.25–0.59)**	< .001
Antenatal screening	**0.15 (0.09–0.25)**	< .001	**0.24 (0.13–0.42)**	< .001
Patient request	**0.26 (0.16–0.43)**	< .001	**0.28 (0.17–0.46)**	< .001
Other	**0.26 (0.19–0.36)**	< .001	**0.28 (0.2–0.38)**	< .001
Year of diagnosis				
2015–2019	Reference		–	
2010–2014	0.85 (0.67–1.08)	0.181	0.79 (0.6–1.03)	0.083
2005–2009	1.13 (0.84–1.53)	0.416	0.96 (0.67–1.38)	0.843
2000–2004	1.53 (0.99–2.37)	0.056	1.42 (0.86–2.34)	0.166
Region of residence				
Muscat	Reference		–	
Outside Muscat	1.15 (0.91–1.45)	0.243	**1.31 (1.02–1.7)**	**0.038**
Marital status				
Married	Reference		–	
Single	1.13 (0.9–1.42)	0.280	1.04 (0.78–1.37)	0.798
Divorced/widowed	**1.63 (1.11–2.39)**	**0.013**	1.37 (0.89–2.09)	0.152

*CI* confidence interval, *HIV* human immunodeficiency infection, *MSM* men who have sex with men, *OR* odds ratio

### Factors associated with LP

The univariate analysis (Table 3) revealed that the factors which were significantly associated with LP were similar to those predicted AD. The multivariate analysis (Table 3) revealed that male sex compared to female sex (aOR 1.43, 95% CI: 1.02–2.02) was the only factor that significantly increased the odds of LP. In contrast, compared to testing for HIV due to HIV related symptoms, testing for HIV because of patient’s request (aOR: 0.23, 95% CI: 0.14–0.38) or antenatal screening (aOR: 0.21, 95% CI: 0.12–0.36) or being in contact with an HIV positive individual (aOR: 0.44, 95% CI: 0.28–0.71) or “other” reason (aOR: 0.3, 95% CI: 0.21–0.42) significantly decreased the odds of presenting with LP.

**Table 3:** Factors associated with late presentation in Oman, 2000–2019 (*N* = 1418)

Variable	Crude OR (95% CI)	***P*** value	Adjusted OR (95% CI)	***P*** value
Sex				
Female	Reference		–	
Male	**1.71 (1.34–2.18)**	< .001	**1.43 (1.02–2.02)**	0.039
Age at diagnosis category (years)				
13–24	Reference		–	
25–49	1.29 (0.99–1.7)	0.062	1.09 (0.81–1.48)	0.569
≥ 50	**2.92 (1.71–4.96)**	< .001	1.66 (0.91–3.01)	0.098
HIV Risk factor				
Heterosexual	Reference		–	
MSM	1.317 (0.98–1.77)	0.068	1.06 (0.75–1.5)	0.732
Other	0.46 (0.22–0.92)	0.029	0.52 (0.24–1.11)	0.089
Unknown	**1.7 (1.13–2.55)**	0.011	1.07 (0.67–1.71)	0.771
Reason for HIV testing				
HIV-related symptoms	Reference		–	
HIV contact	**0.36 (0.23–0.55)**	< .001	**0.44 (0.28–0.71)**	< .001
Antenatal screening	**0.14 (0.09–0.23)**	< .001	**0.21 (0.12–0.36)**	< .001
Patient request	**0.22 (0.13–0.36)**	< .001	**0.23 (0.14–0.38)**	< .001
Other	**0.28 (0.2–0.4)**	< .001	**0.3 (0.21–0.42)**	< .001
Year of diagnosis				
2015–2019	Reference		–	
2010–2014	1.05 (0.81–1.36)	0.726	0.97 (0.73–1.29)	0.826
2005–2009	1.39 (0.99–1.96)	0.057	1.05 (0.71–1.56)	0.801
2000–2004	1.43 (0.87–2.36)	0.162	1.1 (0.63–1.94)	0.733
Region of residence				
Muscat	Reference		–	
Outside Muscat	1.12 (0.87–1.44)	0.381	1.24 (0.94–1.64)	0.125
Marital status				
Married	Reference		–	
Single	1.08 (0.85–1.38)	0.534	0.93 (0.69–1.27)	0.658
Divorced/widowed	**1.64 (1.04–2.58)**	0.033	1.35 (0.82–2.21)	0.239

*CI* confidence interval, *HIV* human immunodeficiency infection, *MSM* men who have sex with men, *OR* odds ratio

## Discussion

Our data revealed high rates of LP and AD among our cohort; the proportions of both increased with age at diagnosis. Of note, the overall rates of LP and AD and those among heterosexuals modestly declined over time; however, levels of late HIV diagnosis among MSM were unchanged. Male sex, older age, having an “unknown” HIV risk factor, and living outside Muscat were independent predictors of AD. Male sex also independently predicted LP. Testing for HIV due to a patient’s request, antenatal screening, and being in contact with an individual with HIV infection significantly decreased the odds of both AD and LP.

The prevalence of LP and AP we observed in our study was higher than that reported in neighbouring countries. A recent study from Saudi Arabia reported that out of 997 patients diagnosed in 2001–2013, 50 and 28.3% presented to care with a CD4 cell count of < 350 and < 200 cells/mm^3^, respectively [14]. Studies conducted outside the Middle East and North Africa (MENA) region, using the same definitions of LP and AD, revealed similar or higher proportions of LP and AD [15–17]. Data from 17 European countries showed that out of 39,204 persons diagnosed with HIV in 2010–2016, 48.4% were classified as having LP, ranging from 36.9% in Estonia and Ukraine to 64.2% in Poland [15]. Furthermore, a study from China reported that out of 45,118 newly diagnosed patients in 2012–2016, 70.2% had LP, and 45.1% had AD [16].

Consistent with previous studies [18–20], male sex and older age (≥ 50 years) were strong predictors of late HIV diagnosis in our study. A recent meta-analysis of 32 studies revealed that men had higher odds of presenting with AD (pooled aOR 1.73) and LP (pooled aOR 1.38) compared with women [18]. The gender difference we observed in our study could be due to differences in healthcare-seeking behaviour, but the introduction of routine HIV screening in pregnancy in Oman in 2009 might have contributed to the earlier HIV diagnosis in women. Low-risk perception [20] and rapid HIV disease progression in older people [20] might explain, in part, the high proportions of LP and AD we observed in patients aged ≥50 years. Moreover, clinicians might consider HIV testing less often in older patients assuming a low HIV risk among this population.

The current national HIV testing guidelines in Oman [21] recommend facility-based HIV indicator diseases guided testing. However, a large and growing body of evidence suggests this “targeted” approach results in missing HIV cases [22, 23]. Moreover, routine HIV testing, regardless of clinical suspicion or risk, is shown to be feasible, acceptable by both patients and staff, effective in identifying new HIV cases and cost-effective [24–27]. Universal HIV screening in various healthcare settings could significantly reduce the rate of late HIV diagnosis in Oman. However, given the low HIV prevalence in Oman, HIV prevention and testing services targeting key populations and their partners might be more effective. To expand HIV testing in the country, the NAP in Oman has recently conducted several HIV workshops for physicians in primary health care (PHC) and the private sector. Moreover, the NAP published an HIV manual that focuses on HIV testing in PHC in 2019 [28].

A noted strength of this study is its large sample size, which was drawn from a national surveillance system receiving data from all HIV clinics in the country. Furthermore, our results help in filling the knowledge gap about the predictors of late HIV diagnosis in the MENA region, where data on this topic are surprisingly scarce. A limitation of this analysis is that our observed rates of LP and AD might have been underestimated. About a third of patients diagnosed during the study period had no information on CD4 cell count at presentation, with the majority of those belonging to patient subgroups with the highest risk for late HIV diagnosis. Another limitation is that our definition of LP and AD did not include having an AIDS-related condition due to data unavailability. Thus, patients without CD4 count at baseline or with a CD4 count of > 350 cells/mm^3^ who presented with an AIDS-defining disease would not have been counted as LP or AD, which further underestimates our rates of late HIV diagnosis.

## Conclusions

This analysis indicates that a significant proportion of new HIV cases in Oman continue to present late. Overall, rates of LP and AD modestly declined over time; however, this decline was mainly among heterosexuals but not MSM. This study identified patient subgroups at greatest risk of late HIV diagnosis, such as men, older people, and those who test for HIV because of HIV-related symptoms. These findings call for targeted interventions and greater efforts to scale up HIV testing services in Oman, particularly outside Muscat, to enhance ART’s individual and public health benefits through early HIV diagnosis and prompt linkage to care. As monitoring of CD4 cell count is critical to HIV surveillance and optimal clinical management of patients with late HIV diagnosis, regular auditing of CD4 testing at presentation in Oman is needed. In addition, there is an urgent need for further research that explores patient- and programme-specific barriers to early HIV diagnosis in Oman.

## Supplementary Information

**Additional file 1: Supplementary 1**. Sociodemographic and clinical characteristics of Omani patients diagnosed with HIV in 2000–2019 (*N* = 2215) *

## Data Availability

This study used the Ministry of Health HIV Registry data. Data from national registers cannot be shared publicly or by study authors. However, data are available from the Ministry of Health, HIV/STI/Hepatitis Section. Researchers who meet the criteria for access to confidential data can contact the Institutional Data Access at: Director of Communicable Diseases, HIV/STI/Hepatitis Section, Ministry of Health, Muscat, PO. Box 393, Fax: 0096822357540, Tel: 0096822357538, email: hiv_aids@moh.gov.om.

## Abbreviations

aOR: Adjusted odds ratio
AD: Advanced HIV disease
ART: Antiretroviral therapy
CPHL: Central public health laboratory
CI: Confidence interval
cOR: Crude odds ratio
HIV: Human immunodeficiency
IQR: Interquartile range
LP: Late presentation
MSM: Men who have sex with men
MENA: Middle East and North Africa
NAP: National AIDS Programme
PWID: People who inject drug
PHC: Primary health care
VL: Viral load
UNAIDS: Joint United Nations Programme on HIV and AIDS

## References

[1] Croxford S, Kitching A, Desai S, Kall M, Edelstein M, Skingsley A (2017). Mortality and causes of death in people diagnosed with HIV in the era of highly active antiretroviral therapy compared with the general population: An analysis of a national observational cohort. Lancet Public Health.

[2] Brown AE, Kall MM, Smith RD, Yin Z, Hunter A, Hunter A (2012). Auditing national HIV guidelines and policies: The United Kingdom CD4 Surveillance Scheme. Open AIDS J.

[3] Marks G, Crepaz N, Senterfitt JW, Janssen RS (2005). Meta-analysis of high-risk sexual behavior in persons aware and unaware they are infected with HIV in the United States: implications for HIV prevention programs. J Acquir Immune Defic Syndr.

[4] Cohen MS, Chen YQ, McCauley M, Gamble T, Hosseinipour MC, Kumarasamy N, Hakim JG, Kumwenda J, Grinsztejn B, Pilotto JH, Godbole SV, Mehendale S, Chariyalertsak S, Santos BR, Mayer KH, Hoffman IF, Eshleman SH, Piwowar-Manning E, Wang L, Makhema J, Mills LA, de Bruyn G, Sanne I, Eron J, Gallant J, Havlir D, Swindells S, Ribaudo H, Elharrar V, Burns D, Taha TE, Nielsen-Saines K, Celentano D, Essex M, Fleming TR, HPTN 052 Study Team (2011). Prevention of HIV-1 infection with early antiretroviral therapy. N Engl J Med.

[5] Krentz HB, Gill MJ (2012). The direct medical costs of late presentation (<350/mm) of HIV infection over a 15-year period. AIDS Res Treat.

[6] Antinori A, Coenen T, Costagiola D, Dedes N, Ellefson M, Gatell J, Girardi E, Johnson M, Kirk O, Lundgren J, Mocroft A, D'Arminio Monforte A, Phillips A, Raben D, Rockstroh JK, Sabin C, Sönnerborg A, de Wolf F, for the European Late Presenter Consensus working group (2011). Late presentation of HIV infection: a consensus definition. HIV Med.

[7] Deblonde J, De KP, Hamers FF, Fontaine J, Luchters S, Temmerman M (2010). Barriers to HIV testing in Europe: a systematic review. Eur J Pub Health.

[8] Elgalib A, Fidler S, Sabapathy K (2018). Hospital-based routine HIV testing in high-income countries: a systematic literature review. HIV Med.

[9] UNAIDS (2018). Miles to go: global AIDS update 2018.

[10] Ministry of Health Oman. Annual Health Report 2018, Published in 2019, Chapter 1, P. 1–4, https://www.moh.gov.om/en/web/statistics/-/20-59. (Accessed 04 July 2020).

[11] Elgalib A, Shah S, Al-Wahaibi A, Al-Habsi Z, Al-Fouri M, Lau R, et al. The Epidemiology of HIV in Oman, 1984–2018: A Nationwide Study from the Middle East. J Epidemiol Global Health. Available online: 30 January 2020. Available on 10.2991/jegh.k.191208.001. 10(3):222–9. 10.2991/jegh.k.191208.001 PMC7509104 32954713

[12] The Joint United Nations Joint Programme on HIV/AIDS (UNAIDS). AIDSinfo. https://aidsinfo.unaids.org. (Accessed 01 August 2020).

[13] Elgalib A, Shah S, Al-Habsi Z, Al-Fouri M, Lau R, Al-Kindi H (2020). The cascade of HIV care in Oman, 2015–2018: a population-based study from the Middle East. Int J Infect Dis.

[14] Memish ZA, Al-Tawfiq JA, Filemban SM, Qutb S, Fodail A, Ali B (2015). Antiretroviral therapy, CD4, viral load, and disease stage in HIV patients in Saudi Arabia: a 2001-2013 cross-sectional study. J Infect Dev Ctries.

[15] Late Presentation Working Groups in EuroSIDA and COHERE (2020). Estimating the burden of HIV late presentation and its attributable morbidity and mortality across Europe 2010–2016. BMC Infect Dis.

[16] Hu X, Liang B, Zhou C, Jiang J, Huang J, Ning C, Liu J, Zhou B, Zang N, Lai J, Chen R, Liao Y, Pan P, Liu X, Lan G, Pang X, Ye L, Shen Z, Liang H (2019). HIV late presentation and advanced HIV disease among patients with newly diagnosed HIV/AIDS in southwestern China: a large-scale cross-sectional study. AIDS Res Ther.

[17] Tsintsadze M, Sharvadze L, Gabunia P, Dvali N, Abutidze A, Tsertsvadze T (2017). Late presentation of HIV infection in the country of Georgia: 2012-2015. PLoS One.

[18] Jiang H, Yin J, Fan Y, Liu J, Zhang Z, Liu L, Nie S (2015). Gender difference in advanced HIV disease and late presentation according to European consensus definitions. Sci Rep.

[19] Castilla J, Lorenzo JM, Izquierdo A, Lezaun ME, López I, Moreno-Iribas C (2006). Characteristics and trends of newly diagnosed HIV-infections, 2000–2004. Gac Sanit.

[20] Thanawuth N, Chongsuvivatwong V (2008). Late HIV diagnosis and delay in CD4 count measurement among HIV-infected patients in southern Thailand. AIDS Care.

[21] HIV management in Oman. A guide for health care workers, 3rd edition. 2015. Available from: www.moh.gov.om/en/web/directorate-general-of-disease-surveillance-control/resources. (accessed 05 August 2020).

[22] Duffus WA, Weis K, Kettinger L, Stephens T, Albrecht H, Gibson JJ (2009). Risk-based HIV testing in South Carolina health care settings failed to identify the majority of infected individuals. AIDS Patient Care STDs.

[23] Jenkins TC, Gardner EM, Thrun MW, Cohn DL, Burman WJ (2006). Risk-based human immunodeficiency virus (HIV) testing fails to detect the majority of HIV-infected persons in medical care settings. Sex Transm Dis.

[24] Haukoos JS, Hopkins E, Byyny RL (2008). Denver emergency department HIV testing study group. Patient acceptance of rapid HIV testing practices in an urban emergency department: assessment of the 2006 CDC recommendations for HIV screening in health care settings. Ann Emerg Med.

[25] Hill-Tout R, Cormack I, Elgalib A (2016). Routine HIV testing in acute medical admissions in a high prevalence area reduces morbidity and mortality of HIV: a full cycle audit. Int J STD AIDS.

[26] Phillips D, Barbour A, Stevenson J, Draper S, Motazed R, Elgalib A (2014). Implementation of a routine HIV testing policy in an acute medical setting in a UK general hospital: a cross-sectional study. Sex Transm Infect.

[27] Sanders GD, Bayoumi AM, Sundaram V, Bilir SP, Neukermans CP, Rydzak CE, Douglass LR, Lazzeroni LC, Holodniy M, Owens DK (2005). Cost- effectiveness of screening for HIV in the era of highly active antiretroviral therapy. N Engl J Med.

[28] HIV in primary health care manual, Directorate General for Disease surveillance and control, Ministry of Health, Oman, First edition, 2019. www.moh.gov.om/en/web/directorate-general-of-disease-surveillance-control/resources.

