# Supplementary material for: Predictors of late presentation and advanced HIV disease among people living with HIV in Oman (2000–2019)
Source: BMC Public Health. 2021 Nov 6;21:2029. doi: 10.1186/s12889-021-12048-1 (PMC8572420; doi:10.1186/s12889-021-12048-1)
Supplement: Supplementary file 1 — Additional file 1: Supplementary 1. Sociodemographic and clinical characteristics of Omani patients diagnosed with HIV in 2000–2019 (N = 2215) * [file 12889_2021_12048_MOESM1_ESM.docx]

Supplementary 1. Sociodemographic and clinical characteristics of Omani patients diagnosed with HIV in 2000-2019 (N= 2215) *

| **Characteristic** | **Cases with**  **CD4 cell count (n/%)** | **Cases without CD4 cell count (n/%)** |
| --- | --- | --- |
| Total | 1418 (64) | 797 (36) |
| Sex |  |  |
| Male (n= 1654) | 1003 (61) | 651 (39) |
| Female (n= 561) | 415 (74) | 146 (26) |
| Age at diagnosis category (years) |  |  |
| 13-24 (439) | 308 (70) | 131 (30) |
| 25-49 (n= 1500) | 982 (65) | 518 (35) |
| ≥ 50 (n= 276) | 128 (46) | 148 (54) |
| HIV Risk factor |  |  |
| Heterosexual (n= 1335) | 934 (70) | 401 (30) |
| MSM (n= 451) | 296 (66) | 155 (34) |
| Others (n= 69) | 32 (46) | 37 (54) |
| Unknown (n= 360) | 156 (43) | 204 (57) |
| Reason for HIV testing (N= 1236) |  |  |
| HIV-related symptoms (n= 484) | 417 (86) | 67 (14) |
| Contact screening (n= 154) | 143 (83) | 11 (17) |
| Antenatal care (n= 117) | 108 (92) | 9 (8) |
| Client initiated testing (n= 111) | 90 (81) | 21 (19) |
| Others (n= 370) | 314 (85) | 56 (15) |
| Year of diagnosis |  |  |
| 2000-2004 (n= 399) | 95 (24) | 304 (76) |
| 2005-2009 (n= 493) | 239 (48) | 254 (52) |
| 2010-2014 (n= 603) | 481 (80) | 122 (20) |
| 2015-2019 (n= 720) | 603 (84) | 117 (16) |
| Region of residence |  |  |
| Muscat (n= 616) | 392 (64) | 224 (36) |
| Outside Muscat (n= 1599) | 1026 (64) | 573 (36) |
| Marital status (N=2103) |  |  |
| Married (n= 1088) | 728 (67) | 360 (37) |
| Single (n= 806) | 519 (64) | 287 (36) |
| Divorced/widowed (n= 209) | 124 (59) | 85 (41) |

HIV, human immunodeficiency virus; MSM, men who have sex with men.

*Unless otherwise stated
